# Oxidative Stress and Redox Signaling in Gastric Cancer: From Mechanisms to Therapeutic Implications

**DOI:** 10.3390/antiox14030258

**Published:** 2025-02-24

**Authors:** Zehua Chen, Jiawu Fan, Xiaolong Chen, Kun Yang, Kui Wang

**Affiliations:** 1 Department of General Surgery and Laboratory of Gastric Cancer, West China School of Basic Medical Sciences & Forensic Medicine, State Key Laboratory of Biotherapy/Collaborative Innovation Center of Biotherapy and Cancer Center, West China Hospital, Sichuan University, Chengdu 610041, China; chenzehuamed@163.com (Z.C.); fanjiawu_scu@163.com (J.F.); huaxichen1987@126.com (X.C.); 2 Gastric Cancer Center, West China Hospital, Sichuan University, Chengdu 610041, China

**Keywords:** redox signaling, oxidative stress, ROS, oxPTMs, gastric cancer, natural products

## Abstract

Oxidative stress, which is characterized by an imbalance between reactive oxygen species (ROS) production and antioxidant defenses, has critical roles in the initiation, progression, and treatment of gastric cancer. On the one hand, an excessive ROS accumulation induces oxidative damage and cancer cell death. On the other hand, moderate levels of ROS cause genetic mutations and dysregulation of signaling pathways to promote proliferation, inflammation, angiogenesis, and metastasis in gastric cancer. Notably, emerging evidence has revealed that ROS also mediate oxidative post-translational modifications (oxPTMs) of redox-sensitive proteins, which can directly affect protein functions and regulate redox signaling in cancer cells. Therefore, elucidating the regulatory mechanisms of oxidative stress and redox signaling in gastric cancer holds great promise to identify novel therapeutic targets or redox-targeting strategies. This review will summarize the mechanisms of oxidative stress in regulating the hallmarks of gastric cancer and highlight the roles of ROS-mediated oxPTMs in gastric cancer. In addition, we will discuss emerging strategies targeting oxidative stress for the treatment of gastric cancer, with an emphasis on the use of bioactive natural products and nanomaterials.

## 1. Introduction

Gastric cancer remains a global public health challenge, with approximately 1,000,000 new diagnoses each year and up to 700,000 cancer-related deaths worldwide [1,2]. The main risk factors for gastric cancer include *Helicobacter pylori* infection [3], smoking [4], obesity [5], metabolic disorders [6], and dietary factors [7,8]. Currently, the comprehensive management of gastric cancer is becoming increasingly standardized. Surgical intervention is considered the cornerstone of treatment [9,10,11,12]. However, gastric cancer exhibits highly invasive and metastatic characteristics, leading to rapid disease progression. Most gastric cancer patients are diagnosed at advanced stages, thus losing the opportunity for surgery [13,14]. In this regard, drug therapy offers hope for these patients. Notably, some patients develop resistance, which limits the efficacy of chemotherapy and targeted drugs [15,16,17]. Therefore, a thorough understanding of the molecular mechanisms underlying the onset and progression of gastric cancer is essential to enable the development of novel therapeutic strategies.

Reactive oxygen species (ROS) refer to highly reactive oxygen-containing molecules, such as hydrogen peroxide (H_2_O_2_), superoxide anion, and hydroxyl radicals. Cancer cells often exhibit higher levels of ROS compared with normal counterparts. To circumvent ROS-mediated oxidative damage, cancer cells are equipped with powerful antioxidant systems to eliminate excessive ROS. When the generation of ROS exceeds the intracellular antioxidant capacity, it leads to ROS accumulation and resultant oxidative stress [18,19,20]. Once cells enter a state of oxidative stress but below the cytotoxic threshold, it may contribute to cancer initiation and progression by inducing genetic mutations, the dysregulation of signaling pathways, and inflammatory responses [21]. The transmission of oxidative stress within or between cells is referred to as redox signaling, which is currently a hotspot in redox research [2]. Redox signaling not only has a key role in the maintenance of cellular homeostasis but also broadly regulates numerous physiological and pathological processes, including cancer [22,23,24,25].

Emerging studies have suggested that oxidative stress is closely associated with the occurrence and development of gastric cancer. *Helicobacter pylori* infection, high-fat diets, and oncogenic mutation have been found to drive gastric cancer initiation, at least partially, by promoting ROS production. Once gastric mucosal cells undergo malignant transformation, ROS further accelerate the proliferation and metastasis of gastric cancer through redox signaling functions [26,27]. However, when the ROS accumulation is excessive, it will lead to oxidative damage and even cell demise in gastric cancer. Therefore, oxidative stress is considered as a potential therapeutic target for gastric cancer treatment. Therapeutic strategies targeting oxidative stress to suppress the proliferation and survival of gastric cancer cells have been explored, either by modulating intracellular ROS levels or decreasing antioxidant defenses [28]. Key drug categories include oxidizing agents (cisplatin, etc.), inhibitors of antioxidant systems (peroxiredoxin (PRDX) inhibitors, NADPH oxidase (NOX) inhibitors, etc.), and drugs modulating redox signaling pathways (cepharanthine, etc.) [29,30,31,32,33]. Among them, cisplatin is widely used in clinical practice but faces challenges, including drug resistance, side effects, and the lack of optimized drug delivery systems [34]. Meanwhile, other drugs targeting oxidative stress are still in preclinical studies (nortriptyline, histone deacetylase (HDAC) inhibitors, topotecan, etc.) [35,36,37], or clinical trial stages (multivitamins, etc.) [38]. Hence, gaining a deeper understanding of redox-related biological events and exploring their applications in the precision treatment of gastric cancer may provide more effective therapeutic strategies.

## 2. Oxidative Stress and Redox Signaling

**Figure 1:**
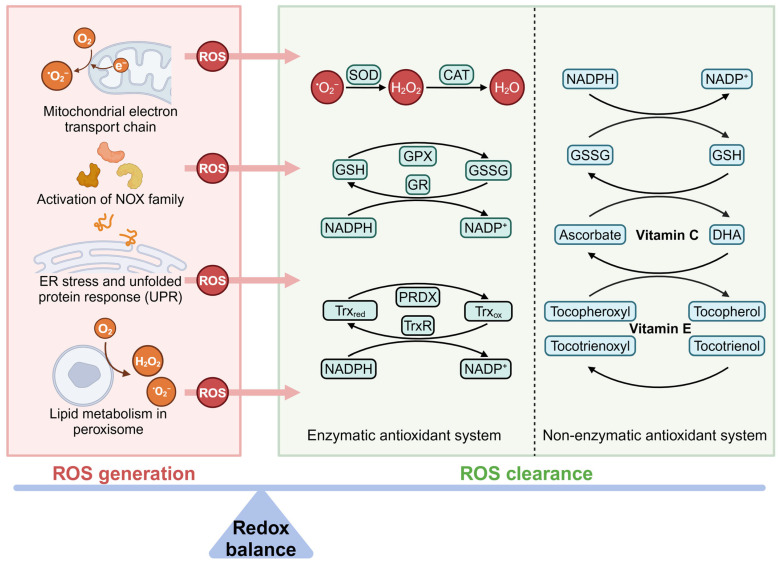
ROS generation and clearance in cells. Reactive oxygen species (ROS) are mainly produced by mitochondria, NADPH oxidases (NOXs), the endoplasmic reticulum (ER), and peroxisomes. For ROS clearance, cells are equipped with enzymatic and non-enzymatic antioxidant systems to maintain intracellular redox balance (created with https://BioRender.com).

The common sources for ROS generation include electron leakage from the mitochondrial respiratory chain, activation of the NOX family, protein folding in the endoplasmic reticulum (ER), and lipid metabolism in the peroxisomes [39]. The antioxidant systems are responsible for eliminating excess ROS to maintain intracellular redox homeostasis. The enzymatic antioxidant systems include superoxide dismutases (SODs), catalase (CAT), glutathione peroxidase (GPx), PRDXs, thioredoxin reductase (TrxR), and others. The non-enzymatic antioxidant systems consist of glutathione (GSH), vitamin C, vitamin E, uric acid, coenzyme Q10, and flavonoids [28] (Figure 1). When the production of ROS is excessive in cells, it can cause damage to proteins, lipids, and DNA, leading to a pathological state characterized by disruption of the internal redox balance [40,41,42].

Early studies indicate that ROS cause oxidative damage to cells. As scientists have deepened this field, increasing evidence suggests that ROS also play an important role in maintaining normal physiological functions [42]. In normal cells, ROS production and the antioxidant systems maintain a dynamic balance. When ROS levels are moderate, they are no longer merely regarded as damaging factors but instead, function in signaling regulation [41]. Numerous signaling pathways, such as mitogen-activated protein kinase (MAPK), PI3K/Akt, and NF-κB, are influenced by ROS [43,44,45]. This allows for the transmission of signals related to cell growth, differentiation, and immune regulation, thereby affecting cell fate [46,47]. Specifically, ROS can directly modify proteins through the reversible or irreversible oxidation of certain amino acid residues, such as cysteine, tyrosine, lysine, and methionine. Common modifications include cysteine oxidation (the formation of disulfide bonds, sulfenic acid, sulfinic acid, sulfonic acid, glutathionylation, etc.), tyrosine nitration, and carbonylation [48]. These oxidative post-translational modifications (oxPTMs) can regulate protein structure or activities, thereby affecting protein function and related biological events [49].

## 3. Redox Insights into the Essential Features of Gastric Cancer

**Figure 2:**
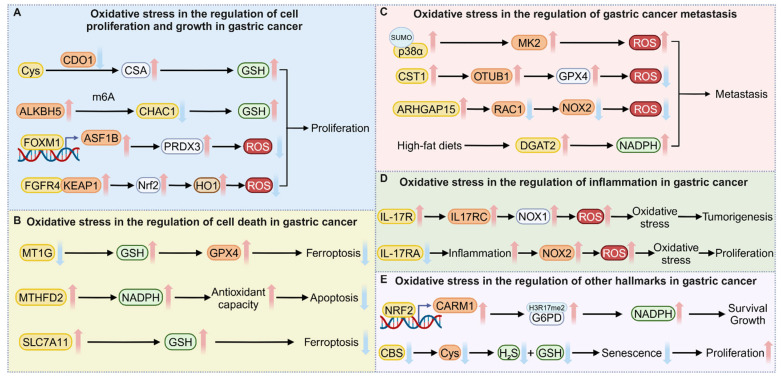
Oxidative stress in the regulation of gastric cancer hallmarks. ROS have crucial roles in the regulation of key cancer characteristics in gastric cancer, including proliferation, cell death (apoptosis and ferroptosis), metastasis, inflammation, epigenetic reprogramming, metabolic rewiring, and senescence. Red arrows indicate promotion or upregulation, while blue arrows indicate inhibition or downregulation. (created with https://BioRender.com).

Gastric cancer, like other cancers, is characterized by sustained proliferative signaling, the evasion of growth suppressors, resistance to cell death, the activation of invasion and metastasis, epigenetic reprogramming, deregulating cellular energetics, resistance to senescence, and other hallmarks [50]. The realization of these hallmarks relies on profound alterations in signaling pathways closely associated with signaling cascades [51]. As an emerging mode of signal transduction, redox signaling has been found to influence the formation of these hallmark traits [52] (Figure 2). Understanding these cancer hallmarks from a redox perspective in gastric cancer not only deepens our knowledge of cancer biology but also provides potential directions for the development of novel diagnostic and therapeutic strategies.

### 3.1. Oxidative Stress in the Regulation of Cell Proliferation and Growth in Gastric Cancer

Oxidative stress plays a critical role in regulating the proliferation of gastric cancer cells. Moderate levels of ROS act as signaling molecules to activate proliferation-related pathways [53]. Given that excessive ROS cause DNA damage and apoptosis [54], gastric cancer cells can maintain ROS below the detrimental threshold by enhancing their antioxidant capacity to sustain rapid growth [55].

GSH is a tripeptide composed of glutamate, cysteine, and glycine, and it serves as a critical antioxidant and detoxifying agent within cells [56]. GSH scavenges ROS and free radicals by its thiol group (-SH) to maintain intracellular redox balance. An elevation of the GSH level can effectively enhance cellular resistance to oxidative stress [57,58]. As an important precursor for GSH synthesis, cysteine plays a vital role in maintaining cellular antioxidant capacity and redox homeostasis. Cysteine dioxygenase type 1 (CDO1) can catalyze the conversion of cysteine to cysteine sulfinic acid, thereby regulating intracellular sulfur metabolism and GSH synthesis. In gastric cancer cells, CDO1 overexpression leads to reduced cysteine levels and decreased GSH synthesis, leading to the accumulation of ROS, the activation of integrated stress response, and the inhibition of cell proliferation [59]. Glutathione-specific gamma-glutamylcyclotransferase 1 (CHAC1) is another enzyme involved in GSH metabolism, capable of degrading GSH to produce 5-oxo-proline and glycine. The RNA demethylase AlkB homolog 5 (ALKBH5), which is highly expressed in gastric cancer, can downregulate CHAC1 by removing m6A modifications. ALKBH5-mediated CHAC1 downregulation enhances GSH levels and strengthens antioxidant capacity, resulting in the increased proliferation of gastric cancer cells [60].

Upregulating antioxidant enzymes, such as SODs, CAT, GPx, and PRDXs, is another important way to promote the antioxidant ability of cells [61]. Among them, PRDXs have been reported to confer resistance to oxidative stress in gastric cancer cells. PRDXs are thioredoxin-dependent peroxidases that efficiently reduces H₂O₂, organic peroxides, and peroxynitrite, thereby scavenging ROS and protecting cells from oxidative damage [54]. For example, anti-silencing function 1B (ASF1B), a highly conserved histone chaperone protein, was found to interact with the transcription factor FOXM1 in gastric cancer cells, resulting in the increased enrichment of FOXM1 in PRDX3 promoter for PRDX3 transcription. This ASF1B-FOXM1-PRDX3 axis confers a powerful antioxidant capacity to detoxify ROS and promote proliferation in gastric cancer cells [33]. Moreover, a newly identified circular RNA, death-inducer obliterator 1 (circDIDO1), encodes 529 aa protein (DIDO1-529aa) and is expressed at low levels in gastric cancer. CircDIDO1 downregulation leads to a decrease in the RING box protein-1 (RBX1)-mediated ubiquitination and degradation of PRDX2, thereby stabilizing PRDX2 protein and enhancing the antioxidant capacity of gastric cancer cells [62].

Notably, the transcription factor nuclear factor erythroid 2-related factor 2 (NRF2) is a master regulator of cellular antioxidant responses [63]. NRF2 can bind to the antioxidant response elements (AREs) to promote the transcription of target genes encoding antioxidant enzymes (such as SODs, CAT, and GPx) and enzymes required for GSH synthesis (such as glutamate–cysteine ligase and glutathione synthetase), thereby enhancing the antioxidant defenses [64]. Under normal conditions, Kelch-like ECH-associated protein 1 (KEAP1) binds to the ETGE and DLG motifs of NRF2 in the cytoplasm through its DGR domain, leading to the cytosolic retention and proteasomal degradation of NRF2. Disrupting this interaction enables NRF2 nuclear translation, which represents a key mechanism for the regulation of NRF2 transcriptional activity in cancer cells [65]. In gastric cancer, overexpressed fibroblast growth factor receptor 4 (FGFR4) forms a complex with p62 and KEAP1, which blocks KEAP1-mediated NRF2 ubiquitination and degradation. This leads to the stabilization and nuclear translocation of NRF2 to counteract ROS elevation in response to *Helicobacter pylori* infection [66]. In addition, family with sequence similarity 117, member B (FAM117B) was reported to interact with the DGR domain of KEAP1, which prevents the ubiquitination degradation of NRF2 and activates the NRF2-mediated transcription of antioxidant genes. FAM117B-mediated NRF2 activation then promotes the growth of gastric cancer cells and reduces their sensitivity to chemotherapeutic drugs [67].

### 3.2. Oxidative Stress in the Regulation of Cell Death in Gastric Cancer

Oxidative stress is essential in regulating various forms of cell death, including apoptosis, ferroptosis, and autophagy. These forms of cell death are closely associated with the progression and treatment response of gastric cancer [68]. However, gastric cancer cells can counteract these death signals, to survive in response to oxidative stress by enhancing antioxidant defenses, reducing ROS production, and maintaining redox balance. These mechanisms include increasing antioxidant generation, regulating iron metabolism, and suppressing autophagy-related degradation [69].

Gastric cancer cells resist oxidative stress-induced ferroptosis by increasing GSH levels. Solute carrier family 7 member 11 (SLC7A11, also termed xCT) is a transmembrane amino acid transporter that belongs to the system Xc− family. It is primarily responsible for transporting cystine from the extracellular space into the cells. Once cystine enters the cells, it is reduced to cysteine, which can combine with glutamate through the catalysis of γ-glutamylcysteine synthetase to form γ-glutamylcysteine. Subsequently, γ-glutamylcysteine, together with glycine, are further catalyzed by glutathione synthetase to form GSH [70]. The ability of gastric cancer cells to resist ferroptosis is due to the high expression of SLC7A11 and the γ-glutamyl-cysteine ligase catalytic subunit (GCLC), which promote GSH synthesis [71,72]. In addition, MT1G, a member of the metallothionein family, is downregulated in gastric cancer. The reduced expression of MT1G leads to elevated intracellular GSH levels, thus enhancing the availability of GSH as a key substrate for GPX4. This increases GPX4 activity and suppresses ferroptosis in gastric cancer cells [73]. Apart from GSH, other antioxidants, such as NADPH, are also critical for gastric cancer cells to counter oxidative stress. Methylene tetrahydrofolate dehydrogenase 2 (MTHFD2) catalyzes the conversion of methylenetetrahydrofolate (MTHF) to formyltetrahydrofolate (Formyl-THF) in mitochondria, while generating NADPH. In gastric cancer, a high expression of MTHFD2 supports cancer cells in resisting oxidative stress-induced cell death by maintaining the NADPH balance and antioxidant capacity [74].

### 3.3. Oxidative Stress in the Regulation of Gastric Cancer Metastasis

ROS, as vital signaling molecules, augment cancer cell invasion and metastatic potential by regulating matrix remodeling, promoting angiogenesis, and enabling immune evasion. However, excessive ROS can trigger cell death and limit metastatic ability. In order to maintain their metastatic characteristics, gastric cancer cells promote ROS production, while simultaneously enhancing ROS clearance to reach a cancer-specific redox balance [75].

A moderate increase in ROS can promote cancer cell metastasis through (1) inducing the epithelial–mesenchymal transition (EMT) of cancer cells to acquire invasive and migratory capabilities and (2) activating signaling pathways (such as MAPK) to facilitate extracellular matrix degradation and cancer cell invasion [76,77]. For example, cystatin SN (CST1) reduces GPX4 ubiquitination by recruiting the deubiquitinating enzyme OTUB1, thereby enhancing GPX4 protein stability and decreasing intracellular ROS levels. This process promotes EMT and the metastasis of gastric cancer cells [78]. P38α, a member of the MAPK kinases, was found to undergo SUMOylation at Lys152, leading to enhanced protein stability and increased nuclear translocation. Nuclear p38α can interact with and activate MAPK-activated protein kinase 2 (MK2) to amplify ROS production. ROS, in turn, stabilize the PIASxα required for the SUMOylation of p38α, therefore forming a positive feedback loop that favors the metastasis of gastric cancer cells [13].

Gastric cancer cells boost their antioxidant capacity to escape oxidative stress-mediated damage, and this mechanism promotes their metastatic potential. It has been reported that Rho GTPase-activating protein 15 (ARHGAP15) is highly expressed in gastric cancer to promote the colonization and metastasis of gastric cancer cells. This is attributable to the ARHGAP15-mediated suppression of RAC1 activity, which leads to NOX2 inactivation and limited ROS production [79]. In addition, high-fat diets upregulate the expression of diacylglycerol O-acyltransferase 2 (DGAT2), which can catalyze the formation of triglycerides to enhance lipid droplet formation and NADPH production. This alleviates oxidative stress and confers anti-apoptotic and adaptive properties to gastric cancer cells, ultimately facilitating peritoneal dissemination and lung metastasis [27].

### 3.4. Oxidative Stress in the Regulation of Inflammation in Gastric Cancer

The interaction between inflammation and oxidative stress in cancer drives cancer’s initiation and progression. Chronic inflammation activates immune cells, such as neutrophils and macrophages, to produce ROS, which cause gene mutations and genomic instability in epithelial cells [75,80]. At the same time, oxidative stress triggers pro-inflammatory signaling pathways to maintain the chronic inflammatory environment [81]. This interplay between inflammation and oxidative stress drives abnormal cell proliferation, inhibits apoptosis, and remodels the cancer microenvironment, ultimately accelerating cancer progression [82,83].

Approximately 16.1% of human cancers are caused by microorganisms, which can induce chronic inflammation and oxidative stress [84]. *Helicobacter pylori* is a Gram-negative, microaerophilic bacterium that infects the stomach and contributes to the development of diseases like gastric cancer. Infection with *Helicobacter pylori* results in a chronic inflammatory state, with persistent oxidative stress in the tissues. Moreover, *Helicobacter pylori* has evolved mechanisms to evade the host’s efforts to eradicate the bacteria. This leads to prolonged inflammation and sustained oxidative stress, which facilitate the development of gastric cancer [85]. Upon infection of the gastric mucosa by *Helicobacter pylori*, the levels of pro-inflammatory cytokines like IL-17A (also known as IL-17) are significantly elevated, forming a chronic inflammatory microenvironment. IL-17A signals through IL-17 receptor A (IL-17RA) and IL-17 receptor C (IL-17RC). Nevertheless, it appears that IL-17RA and IL-17RC play opposite roles in the development of gastric cancer [86,87]. IL-17A promotes ROS production through the IL-17RC/NF-κB/NOX1 pathway. These combined effects of oxidative stress and inflammation disrupt the balance between cell proliferation and apoptosis, which promotes the progression of precancerous lesions such as atrophic gastritis, intestinal metaplasia, and dysplasia, and ultimately triggers cancer [88]. However, it has been found that a deficiency of IL-17RA significantly exacerbates inflammation and immune responses following *Helicobacter pylori* infection, resulting in the formation of lymphoid follicles and an increase in NOX2-mediated ROS accumulation. Compared to wild-type mice, IL-17RA-deficient mice are more prone to developing gastric cancer, with more a severe disease progression [87]. It seems that IL-17RA signaling activates a protective pathway to prevent excessive inflammation and inhibit cancer development.

### 3.5. Oxidative Stress in the Regulation of Other Hallmarks of Gastric Cancer

In addition to the above-mentioned hallmarks, studies have also revealed the role of oxidative stress in regulating other hallmarks of gastric cancer, including epigenetics, metabolic reprogramming, and senescence. For example, a low-glucose environment around gastric cancer cells can upregulate NRF2 expression, which promotes protein arginine methyltransferase 4 (PRMT4, also named CARM1) transcription. The upregulation of PRMT4 mediates the methylation of histone H3 at arginine 17 (H3R17me2) at the promoter of the glucose-6-phosphate dehydrogenase (G6PD) gene, leading to G6PD upregulation. The upregulation of G6PD diverts glucose flux toward the pentose phosphate pathway, to enable nucleotide synthesis (such as ribose-5-phosphate), NADPH production, and the maintenance of redox homeostasis, ultimately supporting the survival and growth of gastric cancer cells [69].

The excessive activation of oncogenic pathways downstream of RAS and PI3K/Akt signaling can induce a senescence-like phenotype. However, cancer cells can circumvent this senescence barrier to facilitate their continuous proliferation. During Akt-induced senescence (AIS), cystathionine β-synthase (CBS) is upregulated and exogenous cysteine uptake is increased, resulting in the enhanced production of hydrogen sulfide (H₂S) and GSH to stabilize the senescent state. In gastric cancers or other cancers dependent on PI3K/Akt signaling, CBS is often epigenetically silenced or downregulated, thus preventing cells from senescence maintenance and regaining the proliferative capacity to drive cancer development [89].

## 4. Oxidative Stress-Mediated oxPTMs in Gastric Cancer

OxPTMs refer to the chemical oxidative modifications of specific amino acid residues (such as cysteine, methionine, and tyrosine) in proteins, which are induced by oxidative agents like ROS. The common types of oxPTMs occur at the active cysteines of proteins, which include sulfenylation, sulfinylation, and sulfonylation, glutathionylation, and the formation of disulfide bonds. Other oxPTMs include methionine oxidation, tyrosine nitration, and the covalent binding of lipid peroxides [90]. Given their capacity for directly regulating protein structure and function, oxPTMs represent an important mechanism for cells to directly respond to oxidative stress and have critical roles in regulating multiple biological events and cancer progression [91].

It has been reported that oxidative stress caused by *Helicobacter pylori* infection can oxidatively modify various proteins in gastric epithelial cells. A recent study used stable isotope labeling by amino acids in cell culture (SILAC) to identify oxidized cysteine residues in *Helicobacter pylori*-infected human gastric adenocarcinoma AGS cells. The researchers employed isoTOP-ABPP to label oxidized thiols in cell lysates and analyzed the differences between *Helicobacter pylori*-infected and uninfected cells using quantitative mass spectrometry (LC-MS/MS). To eliminate the interference of post-transcriptional effects, they combined changes in protein abundance to select cysteines that were directly oxidized. As a result, this study identified eight proteins with cysteine oxidation following *Helicobacter pylori* infection. Among them, legumain, an asparagine-specific endopeptidase, has been shown to promote cancer by modulating the cancer immune microenvironment [92,93,94]. Legumain primarily exists in its inactive form (known as prolegumain), and it can be activated through autocatalytic cleavage to form the mature enzyme. Following *Helicobacter pylori* infection, the Cys219 of legumain undergoes sulfenylation, which inhibits its processing and enzymatic activity. This leads to more prolegumain being secreted into the extracellular space through a ubiquitin-dependent mechanism. Although the oncogenic function of legumain relies on its enzymatic activity, it is intriguing that the Cys219 sulfenylation of legumain induced by *Helicobacter pylori* infection can still promote cancer. The reason may be that after prolegumain is secreted into the extracellular space, it is processed into mature legumain by the acidic environment outside the cells, which then exerts its cancer-promoting effects [95]. In addition to legumain, this study also identified other proteins whose cysteine residues can be directly oxidized during *Helicobacter pylori* infection, including RPL23, TXNDC5, etc. This research undoubtedly provides a solid foundation and valuable insight for future investigations into the roles of oxPTMs in *Helicobacter pylori* infection and gastric cancer progression.

## 5. Targeting Oxidative Stress for the Treatment of Gastric Cancer

Currently, the treatment of advanced gastric cancer primarily relies on chemotherapy, but it still has several drawbacks, such as drug resistance and side effects [96]. During the past decades, due to their multi-target effects, low toxicity, and immune regulatory properties, natural products have gradually attracted much attention as complementary or alternative therapies for cancer treatment, including gastric cancer [97]. However, some natural products have poor stability and low bioavailability. These drawbacks can be addressed by recent advances in nanotechnology, which allow for encapsulation with nanomaterials to improve stability, enhance bioavailability, and enable targeted delivery. Moreover, nanomaterials can directly induce ROS production to exert anticancer effects [98], such as in photodynamic therapy (PDT) and sonodynamic therapy (SDT) [99]. These nanomaterials have a high drug-loading efficiency, prolonged blood circulation, the ability to capture cancer cells, unique cell uptake mechanisms, efficient photothermal conversion, and material adjustability [100].

### 5.1. Natural Products Modulating Oxidative Stress for Gastric Cancer Treatment

It has been reported that various natural compounds induce cell death and inhibit the proliferation and migration of cancer cells through multiple mechanisms, including reducing cellular antioxidant capacity and elevating ROS production [101]. Among them, targeting antioxidant enzymes by natural products to lower the antioxidant capacity and promote the apoptosis of gastric cancer cells has been explored for gastric cancer treatment. For instance, PRDXs, a class of important antioxidant enzymes, prevent oxidative damage by reducing H₂O₂ and organic peroxides [102,103]. Triptolide, a bioactive compound extracted from the herb *Tripterygium wilfordii*, has been found to covalently bind to PRDX2. The binding of triptolide inhibits the antioxidant activity of PRDX2 and causes ROS accumulation, thereby exerting its anticancer effect in gastric cancer [104]. Another natural compound from *Tripterygium wilfordii*, celastrol, exerts similar effects by directly binding to PRDX2 and inhibiting its enzymatic activity [30]. In addition to PRDXs, GPX4 plays an important role in inhibiting lipid peroxidation and ferroptosis through the GSH-dependent pathway. Asiaticoside, a bioactive compound isolated from *Centella asiatica*, downregulates GPX4 expression and suppresses the Wnt/β-catenin signaling pathway. This further enhances ferroptosis and inhibits immune evasion in gastric cancer cells [105]. Quercetin, a natural flavonoid, binds to SLC1A5 and inhibits NRF2 nuclear translocation, leading to the downregulation of SLC7A11 and GPX4, thereby triggering ferroptosis in gastric cancer cells [106]. In addition to reducing antioxidant capacity, promoting ROS generation, such as the activation of NOX4, is another mechanism by which natural products exert anticancer effects in gastric cancer. It was found that the monomethoxyphenyl compound 8-shogaol, derived from *Zingiber officinale Roscoe*, activates NOX4 to increase ROS generation, induce ER stress, and trigger apoptosis in gastric cancer cells. Moreover, 8-shogaol pronouncedly enhances the sensitivity of gastric cancer cells to radiation [107].

An excessive accumulation of ROS can sometimes shift a gene’s function from cancer-promoting to cancer-suppressing. Dehydrocurvularin (DSE2), a fungal-derived macrolide compound, elevates ROS levels and activates PARP-1. Although PARP-1 is involved in DNA damage repair, excessive PARP-1 activation in response to severe DNA damage may trigger the nucleus translocation of apoptosis-inducing factor (AIF), resulting in apoptosis induction in gastric cancer cells [108]. Furthermore, certain natural compounds, such as ginsenoside Rg5, elevate ROS levels and activate the p38 signaling pathway, leading to the apoptosis of gastric cancer cells [109]. Other active plant compounds, such as calycosin and licochalcone A, have also showed favorable anticancer effects in gastric cancer by increasing ROS accumulation, activating p38 signaling, and promoting apoptosis [110,111]. Moreover, oleocanthal, a minor polar compound found in extra-virgin olive oil, elevates ROS levels in gastric cancer cells. Oleocanthal-induced ROS accumulation activates p53 to suppress the cell cycle and growth of gastric cancer cells. In addition, oleocanthal also exhibits synergistic effects when combined with chemotherapeutic agents, including 5-fluorouracil, paclitaxel, and cisplatin [112].

Some natural products also inhibit the signaling pathways that promote cancer growth by stimulating ROS accumulation. For example, magnoflorine, an alkaloid isolated from *Coptis chinensis*, induces autophagic cell death in gastric cancer via the ROS-mediated inhibition of the Akt signaling pathway [113]. Galangin, a flavonoid derived from *Alpinia officinarum* and other members of the ginger family, inhibits the STAT3 pathway and increases intracellular ROS levels [114]. Ginkgolic acid, derived from ginkgo seed coats, has been reported to elevate ROS levels and inhibit the STAT3/JAK2 signaling pathway, thereby promoting the apoptosis of gastric cancer cells [115].

Natural products can also exert anticancer effects through ROS-modulated metabolic reprogramming in gastric cancer cells. Pyruvate dehydrogenase kinase 1 (PDHK1) phosphorylates and inhibits the activity of pyruvate dehydrogenase (PDH). PDHK1-mediated PDH inactivation prevents the entry of pyruvate into mitochondria for aerobic oxidative phosphorylation (OXPHOS), resulting in increased glycolytic flux [116]. Helichrysetin, a flavonoid compound, was found to induce ROS accumulation and suppress the c-Myc/PDHK1 axis. Helichrysetin-mediated PDHK1 downregulation promotes mitochondrial OXPHOS and suppresses glycolysis, leading to the decreased growth of gastric cancer cells [117]. Isoliquiritigenin, another natural flavonoid, can inhibit the expression of c-Myc and HIF-1α by stimulating ROS accumulation in gastric cancer cells, resulting in reduced GLUT4 expression and limited glucose uptake. In addition, isoliquiritigenin induces a collapse in energy metabolism through blocking the PDHK1/PGC-1α axis, ultimately inhibiting gastric cancer growth [118] (Table 1).

### 5.2. Nanomaterials Modulating Oxidative Stress for Gastric Cancer Treatment

Although natural products exhibit potent anticancer effects in gastric cancer, some of them have limitations that restrict their clinical applications, such as low solubility and poor biocompatibility. Fortunately, nanomaterial encapsulation can effectively address these challenges. For example, flavonoids and polyphenols are of high abundance in *Cirsium japonicum* (CJ) and are capable of reducing metal ions to act as capping agents. Therefore, the ethanol extract of CJ was used as a raw material to synthesize a novel type of gold nanoparticles (AuNPs), termed CJ-mediated AuNPs (CJ-AuNPs). CJ-AuNPs can induce the accumulation of mitochondrial ROS, Fe^2+^, and lipid peroxides to disrupt GPX4-dependent antioxidant capacity, leading to mitochondrial damage and apoptosis in gastric cancer cells [119]. Ursolic acid exhibits effective anticancer activity against gastric cancer, but its low solubility and poor biocompatibility hinder its therapeutic efficacy and clinical translation. To this end, a novel ROS-sensitive ursolic acid dimer prodrug was developed, which self-assembles into stable nanoparticles in the presence of surfactants. This nanoparticle achieves a high drug-loading capacity and can rapidly and selectively cleave the dimer into active ursolic acid molecules under oxidative stress conditions. Additionally, surface modification with internalized RGD (iRGD) increases the cancer targeting of this nanoparticle [120]. Icaritin, another natural product with anti-gastric cancer activity, exhibits limited bioavailability due to a poor internal permeability and efflux mediated by transporters. To address this disadvantage, icaritin was loaded into poly lactic-co-glycolic acid (PLGA), a biodegradable and biocompatible material with a small size, to generate PLGA@Icariin nanopartciles. PLGA@Icariin promotes ROS generation in gastric cancer cells, resulting in a significant loss of mitochondrial membrane potential and excessive production of oxidative mitochondrial DNA (Ox-mitoDNA). This further allows the release of damage-associated molecular patterns for the induction of immunogenic cell death [121].

Nanomaterials not only have the ability to carry drugs and address issues such as low bioavailability, but they also possess certain physicochemical properties that help deliver drugs accurately to specific tumor environments. The cancer microenvironment of gastric cancer cells has a lower pH compared to other types of cancers. This unique characteristic provides a favorable condition for the use of nanomaterials to achieve precise drug delivery for gastric cancer treatment. For example, novel upconversion nanoparticles (UCNPs) loaded with melatonin are encapsulated in an NIR-responsive biopolymer chitosan shell, which exhibits a good melatonin drug release at pH 5.0. This can increase ROS levels and activate the PI3K/Akt/mTOR signaling pathway, resulting in autophagic cell death in gastric cancer cells [122]. In another study, triple-helix β-glucan extracted from *Dictyophora rubrovolvata* (DRP) was loaded with doxorubicin (DRP-Dox) to achieve acid-triggered and sustained drug delivery. DRP-Dox reduces ROS production, alters the mitochondrial membrane potential, and ameliorates inflammation. This further alleviates gastric mucosal injury and ultimately reduces the precancerous lesions of gastric cancer [123].

In addition, nanomaterials can also be applied to hyperthermic intraperitoneal chemotherapy (HIPEC) for gastric cancer therapy. By combining high temperatures with chemotherapy drugs, which are directly infused into the peritoneum for the treatment of peritoneal metastatic tumors, HIPEC has been evidenced to extend patient survival after cytoreductive surgery [124]. However, tumor cells treated with HIPEC tend to develop heat resistance by overexpressing heat shock proteins (HSPs). Recently, a novel bifunctional nano-inhibitor without a carrier has been developed for the management of the peritoneal metastasis of gastric cancer in HIPEC treatment. This nanomedicine strategy not only directly inhibits HSP90 by lowering intracellular ATP levels and disrupting the HSP90 chaperone cycle, but it also synergistically induces ROS accumulation under high-temperature conditions, causing oxidative damage to cancer cells. Moreover, oxidative stress induces pyroptosis by enhancing caspase 1 expression and activating gasdermin D cleavage, thus significantly improving therapeutic efficacy [125].

Apart from utilizing pH and temperature, nanomaterials themselves can directly elevate ROS levels to kill tumor cells, including in chemodynamic therapy (CDT), photothermal therapy (PTT), and photodynamic therapy (PDT). CDT based on Fenton chemistry has been used as a ferroptosis-targeting strategy for cancer therapy, while PTT can enhance the efficiency of CDT. A platform based on PEGylated manganese-doped polydopamine (PDA) nanoparticles, named PEG-PDA@Mn (PP@Mn) NPs, was constructed. PP@Mn NPs promote the production of ROS through a Fenton-like reaction combined with PTT, leading to ferroptosis in gastric cancer cells. Additionally, under magnetic resonance imaging (MRI) guidance, PP@Mn NPs combined with PTT at the tumor sites exert CDT anticancer effects [126]. CDT utilizes the Fenton reaction, whereas PDT effectively activates photosensitizers and generates ROS through light absorption properties, multi-spectral responses, and other mechanisms. A novel nanoparticle named IRCB@M enhances PDT via a dual effect. Inside the nanocomposite, the photosensitizer (IR-780) and glutaminase inhibitor (CB-839) can self-assemble, followed by being encapsulated by the cancer cell membrane for homologous targeting. IRCB@M reduces the levels of NADPH and reduces GSH by blocking glutamine metabolism, and it amplifies the cytotoxic effects of IR-780-mediated PDT, thereby exerting anti-gastric cancer effect in vivo [127].

### 5.3. Synthetic Compounds Modulating Oxidative Stress for Gastric Cancer Treatment

In addition to natural products and nanomaterials, synthetic and semi-synthetic compounds that harness ROS for anticancer purposes also show promise in gastric cancer therapy. Recent studies have identified compounds capable of inhibiting gastric cancer cell growth by suppressing NRF2. For example, nortriptyline, a commonly used antidepressant, activates Keap1 and consequently inhibits NRF2 activity, resulting in increased ROS levels and the apoptosis of gastric cancer cells. The apoptotic effect initiated by nortriptyline can be partially reversed by N-acetylcysteine (NAC), underscoring the pivotal role of ROS in the anticancer effect of nortriptyline [35]. HDAC inhibitors also exhibit marked anti-gastric cancer effects by inducing oxidative stress and suppressing NRF2 activity [36]. Unlike strategies that directly inhibit antioxidant transcription factors, several compounds reduce GSH abundance in gastric cancer cells to achieve tumor suppression. For example, the topoisomerase I inhibitor topotecan can downregulate the glutamine transporter Alanine-Serine-Cysteine transporter 2 (ASCT2) to limit glutamine uptake and lower intracellular glutamate levels, a vital precursor for GSH synthesis. This diminishes the antioxidant defenses and fosters ROS production, which ultimately causes apoptosis. This study indicates that topotecan exerts its anticancer effects not only via DNA damage but also by triggering ROS-mediated cell death [37]. Although an elevation of ROS levels is considered an effective strategy for treating gastric cancer, ROS accumulation can provoke inflammatory responses that promote tumor development [128]. Consequently, certain drugs have been reported to mitigate ROS levels to restrain ROS-induced inflammation and tumor progression. For instance, ebselen, an organoselenium compound, can restore the expression of GPX2/4 to diminish the ROS accumulation caused by *Helicobacter pylori* infection. Ebselen inhibits the inflammatory response by reducing the production of IL-8. This negative regulatory effect mitigates the oxidative stress damage induced by inflammation. Therefore, ebselen has great potential in the treatment of gastric cancer [129].

## 6. Conclusions

In this review, we summarize the fundamental characteristics of gastric cancer, such as proliferation, resistance to cell death, metastasis, inflammation, epigenetics, metabolic reprogramming, and senescence, from the perspective of oxidative stress and redox signaling. In addition to these basic features, research on oxidative stress related to other hallmarks of gastric cancer is scarce and requires further investigation. We also discuss the significant role of oxPTMs caused by *Helicobacter pylori* in the progression of gastric cancer. Furthermore, we highlight oxidative stress-targeting therapeutic strategies for treating gastric cancer using natural products and nanomaterials. These studies provide new insights into the treatment of gastric cancer by targeting oxidative stress.

Despite the theoretical feasibility of targeting oxidative stress for gastric cancer treatment, several practical challenges remain. First, oxidative stress itself has dual effects: the physiological levels of ROS are necessary for maintaining normal cell function, and thus the suppression of ROS may interfere with normal cell metabolism, leading to side effects. Second, given the complex and variable tumor microenvironment, simply modulating oxidative stress often fails to achieve sustained efficacy, leading to drug resistance or compensatory changes in tumor cells. Third, there are obvious individual differences regarding oxidative stress. Gastric cancer cells in some patients may be less sensitive to ROS-targeting therapy, making the single regulation of ROS less effective. In addition to the differences among patients, ROS levels within the same patient also vary at different times and in different lesion locations. These variations are primarily attributed to the highly dynamic nature of ROS. To overcome these limitations, it may be beneficial to combine oxidative stress-targeting drugs with traditional chemotherapy, immunotherapy, or radiotherapy. In addition, nanotechnology can be used to precisely deliver ROS-modulating agents to the tumor sites to improve drug bioavailability and reduce systemic side effects.

OxPTMs represent a key mechanism by which ROS directly affect protein functions to regulate redox signaling and cell fate [39]. Conventional ROS-modulating approaches in tumor generally operate on a global level. However, such an indiscriminate method can lead to off-target effects or side effects. In this case, targeting the oxPTMs of key proteins is a method of choice for cancer treatment. For example, high-dose vitamin C exhibits anticancer effect in KRAS- and BRAF-mutant colorectal cancer (CRC) cells by promoting ROS accumulation, inducing the S-glutathionylation of GAPDH at Cys152, and inhibiting glycolysis [130]. More recently, the vitamin K precursor, menadione sodium bisulfite, has shown promise in suppressing pancreatic cancer growth by promoting ROS production and subsequent VPS34 oxidation at Cys54 and Cys61. In this process, cysteine oxidation causes VPS34 inactivation and the resultant depletion of PI(3)P on endocytic vesicles, thus triggering triaptosis, a novel form of cell death [131]. These studies indicate two oxPTM-targeting agents with great clinical potential for cancer therapy. While oxPTMs, such as legumain sulfenylation, have been demonstrated as a crucial pathogenic mechanism of *Helicobacter pylori* infection for gastric cancer [95], strategies targeting these oxPTMs remain to be investigated. In addition, whether oxPTMs are related to other pathogenic mechanisms in gastric cancer merits further exploration.

Despite challenges such as the high dynamicity of ROS and significant variations in treatment responses, the modulation of oxidative stress is expected to become an important component of precision medicine for gastric cancer. A deeper understanding of the regulatory mechanisms of oxidative stress and advances in oxidative stress-related technologies are needed to push this research field forward.

## Figures and Tables

**Table 1:** ROS-modulating natural products for gastric cancer treatment.

Compounds	Chemical Structures	Targets/Signaling Pathways	Functions	Refs.
Celastrol	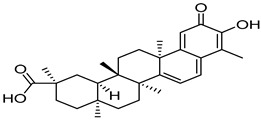	Binding to PRDX2 and inhibiting its enzymatic activity; elevating ROS levels	Inducing apoptosis	[30]
Triptolide	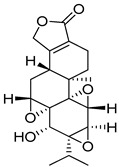	Binding to PRDX2 and inhibiting its enzymatic activity; elevating ROS levels	Inducing apoptosis and cytoprotective autophagy	[104]
Asiaticoside	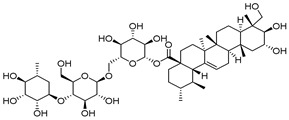	Downregulating GPX4 expression and suppressing the Wnt/β-catenin pathway; elevating ROS levels	Inducing ferroptosis and inhibiting immune evasion	[105]
Quercetin	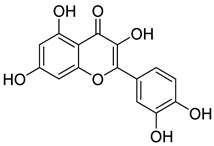	Binding to SLC1A5, inhibiting NRF2, and downregulating xCT/GPX4; elevating ROS levels	Inducing ferroptosis	[106]
8-shogaol	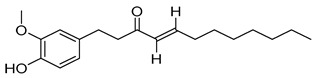	Activating NOX4, elevating ROS levels, and inducing ER stress	Inducing apoptosis and overcoming radioresistance	[107]
Dehydrocurvularin	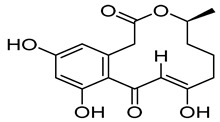	Elevating ROS levels, activating PARP-1, and triggering the nucleus translocation of AIF	Inducing apoptosis	[108]
Ginsenoside Rg5	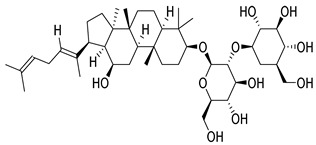	Elevating ROS levels and activating p38 signaling	Inducing apoptosis	[109]
Licochalcone A	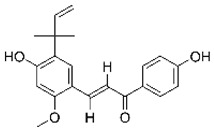	Elevating ROS levels and activating p38 signaling	Inducing apoptosis	[110]
Calycosin	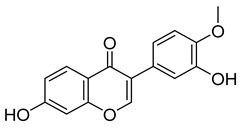	Elevating ROS levels, inhibiting STAT3/NF-κB pathway, and activating p38 signaling	Inducing apoptosis	[111]
Oleocanthal	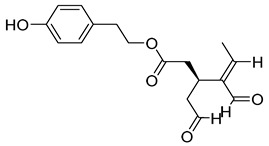	Elevating ROS levels and activating p53 signaling	Suppressing cell cycle and proliferation, sensitizing chemotherapy	[112]
Magnoflorine	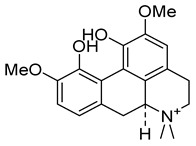	Elevating ROS levels and inhibiting Akt and JNK signaling pathways	Inducing autophagic cell death	[113]
Galangin	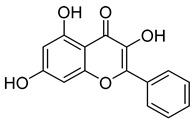	Inhibiting STAT3 pathway, elevating ROS levels, and decreasing NRF2 and NQO-1 expression	Inducing apoptosis and inhibiting proliferation	[114]
Ginkgolic acid	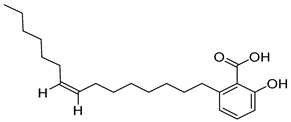	Elevating ROS levels and inhibiting the STAT3/JAK2 signaling pathway	Inducing apoptosis	[115]
Helichrysetin	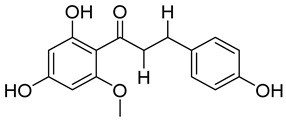	Elevating ROS levels and inhibiting the c-Myc/PDHK1 axis	Inhibiting glycolysis and cell growth	[117]
Isoliquiritigenin	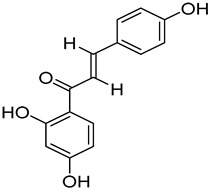	Elevating ROS levels, inhibiting GLUT4 expression, and blocking the PDHK1/PGC-1α axis	Inhibiting glycolysis and cell growth	[118]

## References

[1] Bray F., Laversanne M., Sung H., Ferlay J., Siegel R.L., Soerjomataram I., Jemal A. (2024). Global Cancer Statistics 2022: GLOBOCAN Estimates of Incidence and Mortality Worldwide for 36 Cancers in 185 Countries. CA Cancer J. Clin..

[2] Thrift A.P., Wenker T.N., El-Serag H.B. (2023). Global Burden of Gastric Cancer: Epidemiological Trends, Risk Factors, Screening and Prevention. Nat. Rev. Clin. Oncol..

[3] Lu Y., Xiao F., Wang Y., Wang Z., Liu D., Hong F. (2022). Prevalence of Helicobacter Pylori in Non-Cardia Gastric Cancer in China: A Systematic Review and Meta-Analysis. Front. Oncol..

[4] Li W.-Y., Han Y., Xu H.-M., Wang Z.-N., Xu Y.-Y., Song Y.-X., Xu H., Yin S.-C., Liu X.-Y., Miao Z.-F. (2019). Smoking Status and Subsequent Gastric Cancer Risk in Men Compared with Women: A Meta-Analysis of Prospective Observational Studies. BMC Cancer.

[5] Bae J.-M. (2020). Body Mass Index and Risk of Gastric Cancer in Asian Adults: A Meta-Epidemiological Meta-Analysis of Population-Based Cohort Studies. Cancer Res. Treat..

[6] Zheng J., Gao Y., Xie S.-H., Santoni G., Lagergren J. (2022). Haemoglobin A1c and Serum Glucose Levels and Risk of Gastric Cancer: A Systematic Review and Meta-Analysis. Br. J. Cancer.

[7] Alicandro G., Bertuccio P., Collatuzzo G., Pelucchi C., Bonzi R., Liao L.M., Rabkin C.S., Sinha R., Negri E., Dalmartello M. (2022). The Mediating Role of Combined Lifestyle Factors on the Relationship between Education and Gastric Cancer in the Stomach Cancer Pooling (StoP) Project. Br. J. Cancer.

[8] Morais S., Costa A., Albuquerque G., Araújo N., Pelucchi C., Rabkin C.S., Liao L.M., Sinha R., Zhang Z.-F., Hu J. (2022). Salt Intake and Gastric Cancer: A Pooled Analysis within the Stomach Cancer Pooling (StoP) Project. Cancer Causes Control.

[9] Li S., Yu W., Xie F., Luo H., Liu Z., Lv W., Shi D., Yu D., Gao P., Chen C. (2023). Neoadjuvant Therapy with Immune Checkpoint Blockade, Antiangiogenesis, and Chemotherapy for Locally Advanced Gastric Cancer. Nat. Commun..

[10] Bang Y.-J., Van Cutsem E., Feyereislova A., Chung H.C., Shen L., Sawaki A., Lordick F., Ohtsu A., Omuro Y., Satoh T. (2010). Trastuzumab in Combination with Chemotherapy versus Chemotherapy Alone for Treatment of HER2-Positive Advanced Gastric or Gastro-Oesophageal Junction Cancer (ToGA): A Phase 3, Open-Label, Randomised Controlled Trial. Lancet.

[11] Janjigian Y.Y., Shitara K., Moehler M., Garrido M., Salman P., Shen L., Wyrwicz L., Yamaguchi K., Skoczylas T., Bragagnoli A.C. (2021). Nivolumab plus Chemotherapy versus Chemotherapy as First-Line Treatment for Advanced Gastric Cancer/Gastroesophageal Junction Cancer/Oesophageal Adenocarcinoma (CheckMate 649): A Multicentre, Randomised, Open-Label, Phase 3 Trial. Lancet.

[12] Li G.Z., Doherty G.M., Wang J. (2022). Surgical Management of Gastric Cancer: A Review. JAMA Surg..

[13] Wang Q., Xu C., Fan Q., Yuan H., Zhang X., Chen B., Cai R., Zhang Y., Lin M., Xu M. (2021). Positive Feedback between ROS and Cis-Axis of PIASxα/P38α-SUMOylation/MK2 Facilitates Gastric Cancer Metastasis. Cell Death Dis..

[14] Wang L., Gong W. (2024). NOX4 Regulates Gastric Cancer Cell Invasion and Proliferation by Increasing Ferroptosis Sensitivity through Regulating ROS. Int. Immunopharmacol..

[15] Kim R., An M., Lee H., Mehta A., Heo Y.J., Kim K.-M., Lee S.-Y., Moon J., Kim S.T., Min B.-H. (2022). Early Tumor-Immune Microenvironmental Remodeling and Response to First-Line Fluoropyrimidine and Platinum Chemotherapy in Advanced Gastric Cancer. Cancer Discov..

[16] Peng R., Chen Y., Wei L., Li G., Feng D., Liu S., Jiang R., Zheng S., Chen Y. (2020). Resistance to FGFR1-Targeted Therapy Leads to Autophagy via TAK1/AMPK Activation in Gastric Cancer. Gastric Cancer.

[17] Ouyang S., Li H., Lou L., Huang Q., Zhang Z., Mo J., Li M., Lu J., Zhu K., Chu Y. (2022). Inhibition of STAT3-Ferroptosis Negative Regulatory Axis Suppresses Tumor Growth and Alleviates Chemoresistance in Gastric Cancer. Redox Biol..

[18] Liu Z.-Y., Liu Z.-Y., Lin L.-C., Song K., Tu B., Zhang Y., Yang J.-J., Zhao J.-Y., Tao H. (2024). Redox Homeostasis in Cardiac Fibrosis: Focus on Metal Ion Metabolism. Redox Biol..

[19] Kang Q., Yang C. (2020). Oxidative Stress and Diabetic Retinopathy: Molecular Mechanisms, Pathogenetic Role and Therapeutic Implications. Redox Biol..

[20] Dionísio P.A., Amaral J.D., Rodrigues C.M.P. (2021). Oxidative Stress and Regulated Cell Death in Parkinson’s Disease. Ageing Res. Rev..

[21] Moloney J.N., Cotter T.G. (2018). ROS Signalling in the Biology of Cancer. Semin. Cell Dev. Biol..

[22] Su P., Wang Q., Bi E., Ma X., Liu L., Yang M., Qian J., Yi Q. (2020). Enhanced Lipid Accumulation and Metabolism Are Required for the Differentiation and Activation of Tumor-Associated Macrophages. Cancer Res..

[23] Liu J., Lu W., Shi B., Klein S., Su X. (2019). Peroxisomal Regulation of Redox Homeostasis and Adipocyte Metabolism. Redox Biol..

[24] Morris G., Gevezova M., Sarafian V., Maes M. (2022). Redox Regulation of the Immune Response. Cell Mol. Immunol..

[25] Zhen Z., Ren J., Zhu J. (2024). The Redox Requirement and Regulation during Cell Proliferation. Trends Endocrinol. Metab..

[26] Salvatori S., Marafini I., Laudisi F., Monteleone G., Stolfi C. (2023). Helicobacter Pylori and Gastric Cancer: Pathogenetic Mechanisms. Int. J. Mol. Sci..

[27] Li S., Wu T., Lu Y.-X., Wang J.-X., Yu F.-H., Yang M.-Z., Huang Y.-J., Li Z.-J., Wang S.-L., Huang L. (2020). Obesity Promotes Gastric Cancer Metastasis via Diacylglycerol Acyltransferase 2-Dependent Lipid Droplets Accumulation and Redox Homeostasis. Redox Biol..

[28] Sies H., Jones D.P. (2020). Reactive Oxygen Species (ROS) as Pleiotropic Physiological Signalling Agents. Nat. Rev. Mol. Cell Biol..

[29] Wang Y., Zheng L., Shang W., Yang Z., Li T., Liu F., Shao W., Lv L., Chai L., Qu L. (2022). Wnt/Beta-Catenin Signaling Confers Ferroptosis Resistance by Targeting GPX4 in Gastric Cancer. Cell Death Differ..

[30] Chen X., Zhao Y., Luo W., Chen S., Lin F., Zhang X., Fan S., Shen X., Wang Y., Liang G. (2020). Celastrol Induces ROS-Mediated Apoptosis via Directly Targeting Peroxiredoxin-2 in Gastric Cancer Cells. Theranostics.

[31] Yamamoto T., Nakano H., Shiomi K., Wanibuchi K., Masui H., Takahashi T., Urano Y., Kamata T. (2018). Identification and Characterization of a Novel NADPH Oxidase 1 (Nox1) Inhibitor That Suppresses Proliferation of Colon and Stomach Cancer Cells. Biol. Pharm. Bull..

[32] Lu Y.-Y., Zhu C.-Y., Ding Y.-X., Wang B., Zhao S.-F., Lv J., Chen S.-M., Wang S.-S., Wang Y., Wang R. (2023). Cepharanthine, a Regulator of Keap1-Nrf2, Inhibits Gastric Cancer Growth through Oxidative Stress and Energy Metabolism Pathway. Cell Death Discov..

[33] Zhao Z., Cai Z., Zhang S., Yin X., Jiang T., Shen C., Yin Y., Sun H., Chen Z., Han J. (2024). Activation of the FOXM1/ASF1B/PRDX3 Axis Confers Hyperproliferative and Antioxidative Stress Reactivity to Gastric Cancer. Cancer Lett..

[34] Li J., Qi F., Su H., Zhang C., Zhang Q., Chen Y., Chen P., Su L., Chen Y., Yang Y. (2022). GRP75-Faciliated Mitochondria-Associated ER Membrane (MAM) Integrity Controls Cisplatin-Resistance in Ovarian Cancer Patients. Int. J. Biol. Sci..

[35] Zhu C., Lu Y., Wang S., Song J., Ding Y., Wang Y., Dong C., Liu J., Qiu W., Qi W. (2024). Nortriptyline Hydrochloride, a Potential Candidate for Drug Repurposing, Inhibits Gastric Cancer by Inducing Oxidative Stress by Triggering the Keap1-Nrf2 Pathway. Sci. Rep..

[36] Lorenz L., Zenz T., Oliinyk D., Meier-Rosar F., Jenke R., Aigner A., Büch T. (2024). Vorinostat Treatment of Gastric Cancer Cells Leads to ROS-Induced Cell Inhibition and a Complex Pattern of Molecular Alterations in Nrf2-Dependent Genes. Pharmaceuticals.

[37] Wang L., Liu Y., Zhao T.-L., Li Z.-Z., He J.-Y., Zhang B.-J., Du H.-Z., Jiang J.-W., Yuan S.-T., Sun L. (2019). Topotecan Induces Apoptosis via ASCT2 Mediated Oxidative Stress in Gastric Cancer. Phytomedicine.

[38] Li Z.-J., Chen W., Jiang H., Li X.-Y., Zhu S.-N., Liu X.-H. (2018). Effects of Postoperative Parenteral Nutrition Enhanced by Multivitamin on Metabolic Phenotype in Postoperative Gastric Cancer Patients. Mol. Nutr. Food Res..

[39] Mu B., Zeng Y., Luo L., Wang K. (2024). Oxidative Stress-Mediated Protein Sulfenylation in Human Diseases: Past, Present, and Future. Redox Biol..

[40] Zhang L., Wang X., Cueto R., Effi C., Zhang Y., Tan H., Qin X., Ji Y., Yang X., Wang H. (2019). Biochemical Basis and Metabolic Interplay of Redox Regulation. Redox Biol..

[41] Lennicke C., Cochemé H.M. (2021). Redox Metabolism: ROS as Specific Molecular Regulators of Cell Signaling and Function. Mol. Cell.

[42] D’Autréaux B., Toledano M.B. (2007). ROS as Signalling Molecules: Mechanisms That Generate Specificity in ROS Homeostasis. Nat. Rev. Mol. Cell Biol..

[43] Cho Y., Kim Y.K. (2024). CARM1 Phosphorylation at S595 by P38γ MAPK Drives ROS-Mediated Cellular Senescence. Redox Biol..

[44] Su X., Shen Z., Yang Q., Sui F., Pu J., Ma J., Ma S., Yao D., Ji M., Hou P. (2019). Vitamin C Kills Thyroid Cancer Cells through ROS-Dependent Inhibition of MAPK/ERK and PI3K/AKT Pathways via Distinct Mechanisms. Theranostics.

[45] Chen L., Lu H., Peng D., Cao L.L., Ballout F., Srirmajayam K., Chen Z., Bhat A., Wang T.C., Capobianco A. (2023). Activation of NOTCH Signaling via DLL1 Is Mediated by APE1-Redox-Dependent NF-κB Activation in Oesophageal Adenocarcinoma. Gut.

[46] Sun L., Wang X., Saredy J., Yuan Z., Yang X., Wang H. (2020). Innate-Adaptive Immunity Interplay and Redox Regulation in Immune Response. Redox Biol..

[47] Diaz-Vivancos P., de Simone A., Kiddle G., Foyer C.H. (2015). Glutathione--Linking Cell Proliferation to Oxidative Stress. Free Radic. Biol. Med..

[48] Antelmann H., Helmann J.D. (2011). Thiol-Based Redox Switches and Gene Regulation. Antioxid. Redox Signal.

[49] García-Giménez J.L., Romá-Mateo C., Pallardó F.V. (2019). Oxidative Post-Translational Modifications in Histones. Biofactors.

[50] Hanahan D. (2022). Hallmarks of Cancer: New Dimensions. Cancer Discov..

[51] Wu H., Xiang Z., Huang G., He Q., Song J., Dou R., Yang C., Wang S., Xiong B. (2023). BGN/FAP/STAT3 Positive Feedback Loop Mediated Mutual Interaction between Tumor Cells and Mesothelial Cells Contributes to Peritoneal Metastasis of Gastric Cancer. Int. J. Biol. Sci..

[52] Wang R., Huang W., Cai K., Xiao S., Zhang W., Hu X., Guo J., Mao L., Yuan W., Xu Y. (2023). FLOT1 Promotes Gastric Cancer Progression and Metastasis through BCAR1/ERK Signaling. Int. J. Biol. Sci..

[53] Glorieux C., Liu S., Trachootham D., Huang P. (2024). Targeting ROS in Cancer: Rationale and Strategies. Nat. Rev. Drug Discov..

[54] Park M.H., Jo M., Kim Y.R., Lee C.-K., Hong J.T. (2016). Roles of Peroxiredoxins in Cancer, Neurodegenerative Diseases and Inflammatory Diseases. Pharmacol. Ther..

[55] Luo Y., Xiang W., Liu Z., Yao L., Tang L., Tan W., Ye P., Deng J., Xiao J. (2022). Functional Role of the SLC7A11-AS1/xCT Axis in the Development of Gastric Cancer Cisplatin-Resistance by a GSH-Dependent Mechanism. Free Radic. Biol. Med..

[56] Niu B., Liao K., Zhou Y., Wen T., Quan G., Pan X., Wu C. (2021). Application of Glutathione Depletion in Cancer Therapy: Enhanced ROS-Based Therapy, Ferroptosis, and Chemotherapy. Biomaterials.

[57] Lapenna D. (2023). Glutathione and Glutathione-Dependent Enzymes: From Biochemistry to Gerontology and Successful Aging. Ageing Res. Rev..

[58] Wang K., Luo L., Fu S., Wang M., Wang Z., Dong L., Wu X., Dai L., Peng Y., Shen G. (2023). PHGDH Arginine Methylation by PRMT1 Promotes Serine Synthesis and Represents a Therapeutic Vulnerability in Hepatocellular Carcinoma. Nat. Commun..

[59] Ma G., Zhao Z., Qu Y., Cai F., Liu S., Liang H., Zhang R., Deng J. (2022). Cysteine Dioxygenase 1 Attenuates the Proliferation via Inducing Oxidative Stress and Integrated Stress Response in Gastric Cancer Cells. Cell Death Discov..

[60] Chen C., Zhai E., Liu Y., Qian Y., Zhao R., Ma Y., Liu J., Huang Z., Chen J., Cai S. (2023). ALKBH5-Mediated CHAC1 Depletion Promotes Malignant Progression and Decreases Cisplatin-Induced Oxidative Stress in Gastric Cancer. Cancer Cell Int..

[61] Ghoneum M.H., Badr El-Din N.K., Abdel Fattah S.M., Pan D., Tolentino L. (2015). Hydroferrate Fluid, MRN-100, Provides Protection against Chemical-Induced Gastric and Esophageal Cancer in Wistar Rats. Int. J. Biol. Sci..

[62] Zhang Y., Jiang J., Zhang J., Shen H., Wang M., Guo Z., Zang X., Shi H., Gao J., Cai H. (2021). CircDIDO1 Inhibits Gastric Cancer Progression by Encoding a Novel DIDO1-529aa Protein and Regulating PRDX2 Protein Stability. Mol. Cancer.

[63] Torrente L., DeNicola G.M. (2022). Targeting NRF2 and Its Downstream Processes: Opportunities and Challenges. Annu. Rev. Pharmacol. Toxicol..

[64] Adinolfi S., Patinen T., Jawahar Deen A., Pitkänen S., Härkönen J., Kansanen E., Küblbeck J., Levonen A.-L. (2023). The KEAP1-NRF2 Pathway: Targets for Therapy and Role in Cancer. Redox Biol..

[65] Dinkova-Kostova A.T., Copple I.M. (2023). Advances and Challenges in Therapeutic Targeting of NRF2. Trends Pharmacol. Sci..

[66] Soutto M., Zhang X., Bhat N., Chen Z., Zhu S., Maacha S., Genoula M., El-Gazzaz O., Peng D., Lu H. (2024). Fibroblast Growth Factor Receptor-4 Mediates Activation of Nuclear Factor Erythroid 2-Related Factor-2 in Gastric Tumorigenesis. Redox Biol..

[67] Zhou Y., Chen Y., Shi Y., Wu L., Tan Y., Li T., Chen Y., Xia J., Hu R. (2023). FAM117B Promotes Gastric Cancer Growth and Drug Resistance by Targeting the KEAP1/NRF2 Signaling Pathway. J. Clin. Investig..

[68] Stockwell B.R. (2022). Ferroptosis Turns 10: Emerging Mechanisms, Physiological Functions, and Therapeutic Applications. Cell.

[69] Ping M., Li G., Li Q., Fang Y., Fan T., Wu J., Zhang R., Zhang L., Shen B., Guo J. (2024). The NRF2-CARM1 Axis Links Glucose Sensing to Transcriptional and Epigenetic Regulation of the Pentose Phosphate Pathway in Gastric Cancer. Cell Death Dis..

[70] Yan Y., Teng H., Hang Q., Kondiparthi L., Lei G., Horbath A., Liu X., Mao C., Wu S., Zhuang L. (2023). SLC7A11 Expression Level Dictates Differential Responses to Oxidative Stress in Cancer Cells. Nat. Commun..

[71] Ni H., Qin H., Sun C., Liu Y., Ruan G., Guo Q., Xi T., Xing Y., Zheng L. (2021). MiR-375 Reduces the Stemness of Gastric Cancer Cells through Triggering Ferroptosis. Stem Cell Res. Ther..

[72] Yang Z., Zou S., Zhang Y., Zhang J., Zhang P., Xiao L., Xie Y., Meng M., Feng J., Kang L. (2023). ACTL6A Protects Gastric Cancer Cells against Ferroptosis through Induction of Glutathione Synthesis. Nat. Commun..

[73] Meng K., Song J., Qi F., Li J., Fang Z., Song L. (2024). MT1G Promotes Iron Autophagy and Inhibits the Function of Gastric Cancer Cell Lines by Intervening in GPX4/SQSTM1. Sci. Rep..

[74] Mo H.-Y., Wang R.-B., Ma M.-Y., Zhang Y., Li X.-Y., Wen W.-R., Han Y., Tian T. (2024). MTHFD2-Mediated Redox Homeostasis Promotes Gastric Cancer Progression under Hypoxic Conditions. Redox Rep..

[75] Chen A., Huang H., Fang S., Hang Q. (2024). ROS: A “Booster” for Chronic Inflammation and Tumor Metastasis. Biochim. Biophys. Acta Rev. Cancer.

[76] Chatterjee R., Chatterjee J. (2020). ROS and Oncogenesis with Special Reference to EMT and Stemness. Eur. J. Cell Biol..

[77] Bae S., Lim J.W., Kim H. (2021). β-Carotene Inhibits Expression of Matrix Metalloproteinase-10 and Invasion in Helicobacter Pylori-Infected Gastric Epithelial Cells. Molecules.

[78] Li D., Wang Y., Dong C., Chen T., Dong A., Ren J., Li W., Shu G., Yang J., Shen W. (2023). CST1 Inhibits Ferroptosis and Promotes Gastric Cancer Metastasis by Regulating GPX4 Protein Stability via OTUB1. Oncogene.

[79] Zhang F.-F., Jiang C., Jiang D.-P., Cui Y.-Z., Wang X.-Y., Sun L.-Z., Chen M., Lam K.-O., Wu S.-Y., Verhoeft K. (2023). ARHGAP15 Promotes Metastatic Colonization in Gastric Cancer by Suppressing RAC1-ROS Pathway. PLoS Genet..

[80] El-Kenawi A., Ruffell B. (2017). Inflammation, ROS, and Mutagenesis. Cancer Cell.

[81] Shimada K., Crother T.R., Karlin J., Dagvadorj J., Chiba N., Chen S., Ramanujan V.K., Wolf A.J., Vergnes L., Ojcius D.M. (2012). Oxidized Mitochondrial DNA Activates the NLRP3 Inflammasome during Apoptosis. Immunity.

[82] Kim S.S., Ruiz V.E., Carroll J.D., Moss S.F. (2011). Helicobacter Pylori in the Pathogenesis of Gastric Cancer and Gastric Lymphoma. Cancer Lett..

[83] Reuter S., Gupta S.C., Chaturvedi M.M., Aggarwal B.B. (2010). Oxidative Stress, Inflammation, and Cancer: How Are They Linked?. Free Radic. Biol. Med..

[84] de Martel C., Ferlay J., Franceschi S., Vignat J., Bray F., Forman D., Plummer M. (2012). Global Burden of Cancers Attributable to Infections in 2008: A Review and Synthetic Analysis. Lancet Oncol..

[85] Wang S., Chen Z., Zhu S., Lu H., Peng D., Soutto M., Naz H., Peek R., Xu H., Zaika A. (2020). PRDX2 Protects against Oxidative Stress Induced by H. Pylori and Promotes Resistance to Cisplatin in Gastric Cancer. Redox Biol..

[86] Chandra V., Li L., Le Roux O., Zhang Y., Howell R.M., Rupani D.N., Baydogan S., Miller H.D., Riquelme E., Petrosino J. (2024). Gut Epithelial Interleukin-17 Receptor A Signaling Can Modulate Distant Tumors Growth through Microbial Regulation. Cancer Cell.

[87] Brackman L.C., Jung M.S., Green E.H., Joshi N., Revetta F.L., McClain M.S., Markham N.O., Piazuelo M.B., Scott Algood H.M. (2024). IL-17 Signaling Protects against Helicobacter Pylori-Induced Gastric Cancer. Gut Microbes.

[88] Butcher L.D., den Hartog G., Ernst P.B., Crowe S.E. (2017). Oxidative Stress Resulting From Helicobacter Pylori Infection Contributes to Gastric Carcinogenesis. Cell Mol. Gastroenterol. Hepatol..

[89] Zhu H., Chan K.T., Huang X., Cerra C., Blake S., Trigos A.S., Anderson D., Creek D.J., De Souza D.P., Wang X. (2022). Cystathionine-β-Synthase Is Essential for AKT-Induced Senescence and Suppresses the Development of Gastric Cancers with PI3K/AKT Activation. Elife.

[90] Banks C.J., Andersen J.L. (2019). Mechanisms of SOD1 Regulation by Post-Translational Modifications. Redox Biol..

[91] Roos G., Messens J. (2011). Protein Sulfenic Acid Formation: From Cellular Damage to Redox Regulation. Free Radic. Biol. Med..

[92] Wang H., Chen B., Lin Y., Zhou Y., Li X. (2020). Legumain Promotes Gastric Cancer Progression Through Tumor-Associated Macrophages In Vitro and In Vivo. Int. J. Biol. Sci..

[93] Pang L., Guo S., Khan F., Dunterman M., Ali H., Liu Y., Huang Y., Chen P. (2023). Hypoxia-Driven Protease Legumain Promotes Immunosuppression in Glioblastoma. Cell Rep. Med..

[94] Liu C., Wang J., Zheng Y., Zhu Y., Zhou Z., Liu Z., Lin C., Wan Y., Wen Y., Liu C. (2022). Autocrine Pro-Legumain Promotes Breast Cancer Metastasis via Binding to Integrin Avβ3. Oncogene.

[95] Kovalyova Y., Bak D.W., Gordon E.M., Fung C., Shuman J.H.B., Cover T.L., Amieva M.R., Weerapana E., Hatzios S.K. (2022). An Infection-Induced Oxidation Site Regulates Legumain Processing and Tumor Growth. Nat. Chem. Biol..

[96] Joshi S.S., Badgwell B.D. (2021). Current Treatment and Recent Progress in Gastric Cancer. CA Cancer J. Clin..

[97] Harvey A.L., Edrada-Ebel R., Quinn R.J. (2015). The Re-Emergence of Natural Products for Drug Discovery in the Genomics Era. Nat. Rev. Drug Discov..

[98] Zhu X., Zheng W., Wang X., Li Z., Shen X., Chen Q., Lu Y., Chen K., Ai S., Zhu Y. (2024). Enhanced Photodynamic Therapy Synergizing with Inhibition of Tumor Neutrophil Ferroptosis Boosts Anti-PD-1 Therapy of Gastric Cancer. Adv. Sci..

[99] Yang B., Chen Y., Shi J. (2019). Reactive Oxygen Species (ROS)-Based Nanomedicine. Chem. Rev..

[100] Shin J., Kang N., Kim B., Hong H., Yu L., Kim J., Kang H., Kim J.S. (2023). One-Dimensional Nanomaterials for Cancer Therapy and Diagnosis. Chem. Soc. Rev..

[101] Guo C., Wan L., Li C., Wen Y., Pan H., Zhao M., Wang J., Ma X., Nian Q., Tang J. (2024). Natural Products for Gastric Carcinoma Prevention and Treatment: Focus on Their Antioxidant Stress Actions in the Correa’s Cascade. Phytomedicine.

[102] Kang S.W., Rhee S.G., Chang T.-S., Jeong W., Choi M.H. (2005). 2-Cys Peroxiredoxin Function in Intracellular Signal Transduction: Therapeutic Implications. Trends Mol. Med..

[103] Wu X., Luo L., Wang M., Dong L., Fan J., Zeng Y., Li S., Wang K. (2025). PRDX6 Prevents NNMT Ubiquitination and Degradation as a Nonenzymatic Mechanism to Promote Ovarian Cancer Progression. Adv. Sci..

[104] Chen P., Zhong X., Song Y., Zhong W., Wang S., Wang J., Huang P., Niu Y., Yang W., Ding Z. (2024). Triptolide Induces Apoptosis and Cytoprotective Autophagy by ROS Accumulation via Directly Targeting Peroxiredoxin 2 in Gastric Cancer Cells. Cancer Lett..

[105] Ye C., Yao Z., Wang Y., Zhang C. (2024). Asiaticoside Promoted Ferroptosis and Suppressed Immune Escape in Gastric Cancer Cells by Downregulating the Wnt/β-Catenin Pathway. Int. Immunopharmacol..

[106] Ding L., Dang S., Sun M., Zhou D., Sun Y., Li E., Peng S., Li J., Li G. (2024). Quercetin Induces Ferroptosis in Gastric Cancer Cells by Targeting SLC1A5 and Regulating the P-Camk2/p-DRP1 and NRF2/GPX4 Axes. Free Radic. Biol. Med..

[107] Kim T.W., Lee H.G. (2024). Anti-Inflammatory 8-Shogaol Mediates Apoptosis by Inducing Oxidative Stress and Sensitizes Radioresistance in Gastric Cancer. Int. J. Mol. Sci..

[108] Xu H., Shen X., Li X., Yang X., Chen C., Luo D. (2023). The Natural Product Dehydrocurvularin Induces Apoptosis of Gastric Cancer Cells by Activating PARP-1 and Caspase-3. Apoptosis.

[109] Liu Y., Fan D. (2019). Ginsenoside Rg5 Induces G2/M Phase Arrest, Apoptosis and Autophagy via Regulating ROS-Mediated MAPK Pathways against Human Gastric Cancer. Biochem. Pharmacol..

[110] Hao W., Yuan X., Yu L., Gao C., Sun X., Wang D., Zheng Q. (2015). Licochalcone A-Induced Human Gastric Cancer BGC-823 Cells Apoptosis by Regulating ROS-Mediated MAPKs and PI3K/AKT Signaling Pathways. Sci. Rep..

[111] Zhang Y., Zhang J.-Q., Zhang T., Xue H., Zuo W.-B., Li Y.-N., Zhao Y., Sun G., Fu Z.-R., Zhang Q. (2021). Calycosin Induces Gastric Cancer Cell Apoptosis via the ROS-Mediated MAPK/STAT3/NF-κB Pathway. OncoTargets Ther..

[112] Peri S., Ruzzolini J., Urciuoli S., Versienti G., Biagioni A., Andreucci E., Peppicelli S., Bianchini F., Bottari A., Calorini L. (2022). An Oleocanthal-Enriched EVO Oil Extract Induces the ROS Production in Gastric Cancer Cells and Potentiates the Effect of Chemotherapy. Antioxidants.

[113] Sun X.-L., Zhang X.-W., Zhai H.-J., Zhang D., Ma S.-Y. (2020). Magnoflorine Inhibits Human Gastric Cancer Progression by Inducing Autophagy, Apoptosis and Cell Cycle Arrest by JNK Activation Regulated by ROS. Biomed. Pharmacother..

[114] Liang X., Wang P., Yang C., Huang F., Wu H., Shi H., Wu X. (2021). Galangin Inhibits Gastric Cancer Growth Through Enhancing STAT3 Mediated ROS Production. Front. Pharmacol..

[115] Liang J.-R., Yang H. (2020). Ginkgolic Acid (GA) Suppresses Gastric Cancer Growth by Inducing Apoptosis and Suppressing STAT3/JAK2 Signaling Regulated by ROS. Biomed. Pharmacother..

[116] Golias T., Kery M., Radenkovic S., Papandreou I. (2019). Microenvironmental Control of Glucose Metabolism in Tumors by Regulation of Pyruvate Dehydrogenase. Int. J. Cancer.

[117] Wang P., Jin J.-M., Liang X.-H., Yu M.-Z., Yang C., Huang F., Wu H., Zhang B.-B., Fei X.-Y., Wang Z.-T. (2022). Helichrysetin Inhibits Gastric Cancer Growth by Targeting C-Myc/PDHK1 Axis-Mediated Energy Metabolism Reprogramming. Acta Pharmacol. Sin..

[118] Yu M., Pan Q., Li W., Du T., Huang F., Wu H., He Y., Wu X., Shi H. (2023). Isoliquiritigenin Inhibits Gastric Cancer Growth through Suppressing GLUT4 Mediated Glucose Uptake and Inducing PDHK1/PGC-1α Mediated Energy Metabolic Collapse. Phytomedicine.

[119] Mi X.-J., Park H.-R., Dhandapani S., Lee S., Kim Y.-J. (2022). Biologically Synthesis of Gold Nanoparticles Using Cirsium Japonicum Var. Maackii Extract and the Study of Anti-Cancer Properties on AGS Gastric Cancer Cells. Int. J. Biol. Sci..

[120] Ma J., Chen Y., Liang W., Li L., Du J., Pan C., Zhang C. (2021). ROS-Responsive Dimeric Prodrug-Based Nanomedicine Targeted Therapy for Gastric Cancer. Drug Deliv..

[121] Xiao Y., Yao W., Lin M., Huang W., Li B., Peng B., Ma Q., Zhou X., Liang M. (2022). Icaritin-Loaded PLGA Nanoparticles Activate Immunogenic Cell Death and Facilitate Tumor Recruitment in Mice with Gastric Cancer. Drug Deliv..

[122] Fan Z., Shao Y., Jiang X., Zhou J., Yang L., Chen H., Liu W. (2024). Cytotoxic Effects of NIR Responsive Chitosan-Polymersome Layer Coated Melatonin-Upconversion Nanoparticles on HGC27 and AGS Gastric Cancer Cells: Role of the ROS/PI3K/Akt/mTOR Signaling Pathway. Int. J. Biol. Macromol..

[123] Zhang S., Feng X., Yang S., Shi X., Chen J., Zhu R., Li T., Su W., Wang Y., Cao X. (2024). Acid-Triggered Rattan Ball-like β-Glucan Carrier Embedding Doxorubicin to Synergistically Alleviate Precancerous Lesions of Gastric Cancer via P53 and PI3K Pathways. Int. J. Biol. Macromol..

[124] Bonnot P.-E., Piessen G., Kepenekian V., Decullier E., Pocard M., Meunier B., Bereder J.-M., Abboud K., Marchal F., Quenet F. (2019). Cytoreductive Surgery with or Without Hyperthermic Intraperitoneal Chemotherapy for Gastric Cancer with Peritoneal Metastases (CYTO-CHIP Study): A Propensity Score Analysis. J. Clin. Oncol..

[125] Wang Q., Liu P., Wen Y., Li K., Bi B., Li B.-B., Qiu M., Zhang S., Li Y., Li J. (2023). Metal-Enriched HSP90 Nanoinhibitor Overcomes Heat Resistance in Hyperthermic Intraperitoneal Chemotherapy Used for Peritoneal Metastases. Mol. Cancer.

[126] Chen Z., Li Z., Li C., Huang H., Ren Y., Li Z., Hu Y., Guo W. (2022). Manganese-Containing Polydopamine Nanoparticles as Theranostic Agents for Magnetic Resonance Imaging and Photothermal/Chemodynamic Combined Ferroptosis Therapy Treating Gastric Cancer. Drug Deliv..

[127] Li Z., Li X., Lu Y., Zhu X., Zheng W., Chen K., Liu S., Wu J., Guan W. (2024). Improved Photodynamic Therapy Based on Glutaminase Blockage via Tumor Membrane Coated CB-839/IR-780 Nanoparticles. Small.

[128] Zheng S.-Y., Zhu L., Wu L.-Y., Liu H.-R., Ma X.-P., Li Q., Wu M.-D., Wang W.-J., Li J., Wu H.-G. (2023). Helicobacter Pylori-Positive Chronic Atrophic Gastritis and Cellular Senescence. Helicobacter.

[129] Xu L., Gong C., Li G., Wei J., Wang T., Meng W., Shi M., Wang Y. (2018). Ebselen Suppresses Inflammation Induced by Helicobacter Pylori Lipopolysaccharide via the P38 Mitogen-Activated Protein Kinase Signaling Pathway. Mol. Med. Rep..

[130] Yun J., Mullarky E., Lu C., Bosch K.N., Kavalier A., Rivera K., Roper J., Chio I.I.C., Giannopoulou E.G., Rago C. (2015). Vitamin C Selectively Kills KRAS and BRAF Mutant Colorectal Cancer Cells by Targeting GAPDH. Science.

[131] Swamynathan M.M., Kuang S., Watrud K.E., Doherty M.R., Gineste C., Mathew G., Gong G.Q., Cox H., Cheng E., Reiss D. (2024). Dietary Pro-Oxidant Therapy by a Vitamin K Precursor Targets PI 3-Kinase VPS34 Function. Science.

